# Factors related to reducing free sugar intake among white ethnic adults in the UK: a qualitative study

**DOI:** 10.1038/bdjopen.2017.24

**Published:** 2018-02-09

**Authors:** Said Harith Al Rawahi, Koula Asimakopoulou, Jonathon Timothy Newton

**Affiliations:** 1 Social & Behavioural Sciences Unit, Department of Population and Patient Health, Dental Institute, King's College London, Tower Wing, London, UK

## Abstract

**Objective/Aims:**

To determine the barriers and enablers to behavioural change to reduce free sugar intake related to dental caries in a sample of UK adults who identify their ethnicity as White.

**Materials and methods:**

Qualitative study comprising semi-structured interviews of 27 participants. Interviews were recorded, transcribed and analysed using thematic analysis methods. The Capability-Opportunity-Motivation-Behaviour model (COM-B) and the Theoretical Domains Framework (TDF) were used to guide the derivation of themes.

**Results:**

Data saturation occurred at 27 interviews. The COM-B Model and TDF domains captured various factors that may influence the consumption of free sugar. TDF elements which are reflected in the study are: Knowledge; Psychological skills; Memory, attention, and decision processes; Behavioural regulation; Physical skills; Social influence; Environmental context and resources; Social and professional role and identity; Beliefs about capabilities; Beliefs about consequence; Intentions and goals reinforcement; and Emotions. COM-B Model elements which are reflected in the study are: psychological capabilities, physical capabilities, social opportunities, physical opportunities, reflective motivation, and automatic motivation.

**Discussion and conclusion:**

The COM-B model and TDF framework provided a comprehensive account of the barriers and facilitators of reducing sugar intake among white ethnic groups.

## Introduction

Sugar intake of >10% of the total energy intake per day can lead to a high level of dental caries^1–3^ even with exposure to fluoride.^1,2,4^ Therefore, the World Health Organisation published new guidelines recently for free sugar intake in both adults and children,^5^ and suggested the following recommendations:

- a reduction in free sugar intake during the lifespan of individuals,
- a reduction in free sugar intake to <10% of the total energy intake for both adults and children, and
- a conditional recommendation of a further reduction in free sugar intake to <5% of the total energy intake in these populations.

A daily free sugar intake of <10% of the total energy intake reduces the prevalence of dental caries, and a further reduction in free sugar intake to <5% will even further decrease the risk of dental caries.^1,6^ In addition, based on ecological studies, free sugar intake of <5% of the total energy consumed will also prevent the progression of dental caries long-term.^1^ Free sugar intake of 5% of the total energy consumed is equivalent to 7–8 teaspoons (35 g) of sugar for men and 5–6 teaspoons (25 g) for women.^7^

The rate of free sugar intake among adults in the UK, however, is consistently higher than the WHO recommendation. According to the National Diet and Nutrition Survey UK,^8^ the free sugar intake among adults aged 19–64 years old is 12.1% of the total energy intake, and the free sugar intake for those over 65 years is 11.5% of the total energy intake. In addition, the free sugar intake of most ethnic groups in the UK exceeds the goal of <5% of total energy intake; however, the Defra report^9–15^ indicates that people who identify their ethnicity as White have the highest free sugar intake compared with other ethnic groups in the UK.

Many studies have reported on the factors that contribute to the intake of free sugar, including environmental and context, psychological, physical and social factors. Examples of environmental and context factors include: sources of sugar, prices, food content, availability, accessibility and the advertising of sugary foods, low household income; cultural commitments.^16–24^ Examples of psychological factors include the perception of sugary beverages; individual choice; knowledge level. Examples of physical factors are the intake of fast food and being passive, e.g., watching TV for long period.^17,18,20,21,23–25^ Finally an example of social factor is parental control practices.^20^

In addition, there have been several suggestions for interventions to reduce sugar intake including deliver shocking educational messages,^17^ reducing the availability of beverages,^19^ educational strategies for both children and parents and develop a policy at both the government and school levels to reduce the intake of beverages.^20^

A recent survey conducted in Europe sought to identity consumers' attitudes towards healthy eating.^26^ The participants included 2500 adults (males and females) aged 18–75 years old from five European counties, including the UK. The survey identified factors, such as monitoring sugary food intake, searching for foods with low sugar, individuals' perceptions about artificial sweeteners, and their influence on the choice of buying, as factors related to sugar intake.^26^ While this report highlighted some of the factors that need to be understood in order to encourage individuals to make healthy choices in relation to their sugar intake, the published report did not include details about how the research was conducted, nor how the variables that influence sugar intake were identified.

Much of the previous research in this area has not been informed by a theoretical framework of behaviour change, which is essential in the design of effective behaviour change strategies.^27–30^ Interventions based on such models have been shown to be more effective than non-theory based interventions.^31^ Behaviour change theories and models, such as the theory of planned behaviour and social cognitive theory, have been developed to understand behaviours and achieve behavioural change.^32^ In many cases, however, these theories and models of behavioural change have failed to facilitate change due to major limitations, such as a lack of focus on the circumstances of how a behaviour occurs or a lack of coherence.^33,34^ In contrast, more recently a new model known as the capability-opportunity-motivation-behaviour (COM-B) model has been proposed. The COM-B model aims to understand or analyse behaviours and provide a ‘behavioural diagnosis’.^34^ According to the COM-B model, an individual's behaviour is the result of an interaction between three main conditions: the individual's capability to perform the behaviour, the opportunity that facilitates the behaviour, and the motivation that promotes the behaviour at a given moment.^34–36^ The Theoretical Domains Framework (TDF) is a system that consists of 14 domains (constructs) that can be used along with the COM-B model for further analysis of behaviour.^37^ Both models can be used in conjunction to analyse factors that enhance or prevent the practice of any behaviour.^32^ Both frameworks have been used in different fields related to health including: assessing barriers and enablers to delivery of the Healthy Kids Check;^38^ assessing barriers of doctors toward appropriate prescription of older hospitalised patients;^39^ and development of resource linkage and an IT-enabled health coaching program for disadvantaged Latina moms with recent gestational diabetes.^40^ In these studies, the TDF framework and COM-B Model, have shown to be useful tools in diagnosing behaviour (e.g., identifying barriers and facilitators) and developing interventions that meet the need of individuals in the society.

To date, no studies have employed the COM-B model and/ or TDF to understand the barriers and facilitating factors to the reduction of the intake of free sugar to <5% of the total energy intake among White ethnic adults in the UK. To achieve this a qualitative descriptive method was adopted that provides an in-depth identification of facilitating factors and barriers related to the reduction of free sugar intake to <5% of the total energy intake among White ethnic adults in the UK.

## Research question

What are the potential barriers and facilitators to behavioural change to reduce free sugar intake related to dental caries in a sample of UK adults who identify their ethnicity as White.?

## Materials and Methods

Qualitative data were collected through a series of semi-structured interviews^41^ which were then analysed using Thematic analysis.^42^

### Sample

The sample comprised White ethnic adults aged 18 and over.

For the purposes of this study, the White ethnic adults invited to take part were those individuals who live in the UK, spoke English, and identified themselves as White.

The sample frame for this study was based on the ‘flow population’ method, which involves a process where samples are purposefully selected from a particular site or setting.^43^ In this study, the sample frame was the staff and students of King’s College London, because this location was more practical than other sites in terms of accessibility. Also, students and staff are from different White ethnic background in the UK, possibly leading to a varied sample population.

For this study, purposive sampling was used as the aim of the study is associated with particular ethnicity and age. For purposive sampling in this study, maximum variation and snowballing methods were used, as these methods allow sampling of participants or individuals based on the selection criteria but in a widely varied manner.^43^ The selection of the purposive sampling is based on age and ethnicity criteria.

Participants were recruited through King’s College London based on the sample criteria. Participants who voluntarily agreed to take part in the study were asked to sign the consent form and fill out an application describing their demographic data. In addition, participants were contacted through email and telephone to provide further details about the study and to arrange an interview appointment.

### Procedure and data collection

A topic guide was used to guide the interviews. The objectives of the interviews were provided to each participant at the beginning of each session. Privacy and anonymity was emphasised at the commencement of the interviews. The duration of the interviews ranged from 15 to 90 min. One interviewer conducted all of the interviews, which were audio recorded. Participants were informed about the interview taping both in the consent form and before commencing the interview.

The questions were developed based on established guidelines^36^ and mapped to the COM-B and TDF elements.

### Ethical approval

The study was approved by the King’s College London Research Ethics Committee Reference: LRS15/16 2651. All participants provided written informed consent.

### Monetary rewards

An Amazon voucher valued at £20 was given to participants in appreciation of their time after the interview. The purpose of incentives is to encourage the participants to contribute to the study.

### Data analysis

The data were analysed thematically^42^ based on the categories/themes of the TDF and COM-B model. First, the audio file for each interview was imported into the MAXQDA 12 qualitative analysis software for analysis. Second, a theme guide was developed from the already published definitions of the constructs related to the TDF domains and COM-B model elements. In addition, a general criterion related to sugar intake was considered before codes were collated under the themes of TDF. This stage helped to assign all the themes with factors related to sugar intake. Third, after import of the audio files, each file was converted to a transcript for review by the interviewer (SHAR) to ensure consistency of the transcripts with the audio files. A second investigator (JTN) then reviewed a subsample of transcripts and was in agreement with the interviewer. Fourth, excerpts from the transcripts were coded, and relevant codes were collated under a single subtheme and each subtheme was defined. Fifth, each subtheme was mapped to a domain of the TDF. Sixth, the codes, subthemes, and themes were reviewed three times to ensure the validity of the content of each theme. Review of the coding again took place during the mapping and writing stages.

## Results

### Characteristics of participants

Data saturation occurred at 27 participants. Most were from English, British, Irish, Scottish, and other White ethnic groups. These different categories of white ethnicity are based on ethnicity classifications of Office for National Statistics, UK.^44^ A similar number of males and females and of staff and students were included. Table 1 presents the detailed characteristics of the participants.

### Barriers and facilitating factors

The factors that were reported to influence the sugar intake fitted well within the framework of the TDF themes. No comments could be coded to the TDF Optimism theme, which is associated with the Automatic and Reflection elements of COM-B. The following sections describe some of the facilitators and barriers identified in each theme. Please refer to Supplementary Information 2 for more list of barriers and facilitators to reduce free sugar intake among the white ethnic group in the UK.

### Psychological capabilities

#### Knowledge

In the context of this study, knowledge can be seen as both a facilitator and a barrier to change in terms of sugar intake behaviour. For example, awareness of healthy and unhealthy foods, including drinks and acidic foods, which can cause caries, as well as awareness of the amount of sugar in food and the time to eat sweets can help to increase the possibility of reducing sugar intake. Please refer to quotes number 1–2 in Supplementary Information 1.

However, knowledge can be act as barrier to reduce sugar intake. For example lack of knowledge about the term free sugar and sugar and lack of understanding of the recommended free sugar intake per day. Please refer to quotes number 3-4 in Supplementary Information 1.

#### Psychological skills

The psychological skills identified in this study included the ability to calculate the amount of sugar consumed per day, interpreting the labelling of foods, and assessing the sugar content of foods or meals. Please refer to quotes number 5–6 in Supplementary Information 1.

However, some of the participants stated that they do not perform sugar assessment when they eat meals. This can act as a barrier towards reducing free sugar intake. Please refer to quote number 7 in Supplementary Information 1.

### Memory, attention, and decision processes

Memory, attention, and decision processes include remembering sugar intake advice and the amount of sugar in foods or meals and choosing food with lower sugar or natural sugar increases the possibility of reducing free sugar intake. Please refer to quotes number 8, 9, 10 in Supplementary Information 1.

In contrast, memory, attention, and decision processes can also act as barriers towards change. For example, some participants indicated that they do not pay attention to or are less focused on sugar intake and identifying sugar content in foods. Instead, attention is paid to food that has been reduced in price or is part of a special offer. Also, some of these participants indicated that when they eat sweet food they think of their body weight rather than their teeth and other participants indicated that they prioritise taste over health. Moreover, some of the participants prioritise frequency of eating free sugar over the amount of free sugar and other participants choose food with low calories instead of food low free sugar. In addition, some of the participants raised concerns about their trust of the labelling system and stating that these concerns influence their decision process to reduce sugar intake. Please refer to quotes number 11–19 in Supplementary Information 1.

### Behavioural regulation

Behavioural regulation factors play an important role in minimising sugar intake. Setting of an action plan for daily sugar intake, selecting small size portions of sweets, and prioritising fresh food can help in reducing sugar intake. Please refer to quotes number 20–21 in Supplementary One. Some participants said that they think first their body weight and fitness then dental health. Also, considering food calories first then other element of food labelling can act as a barrier, because not all low calorie food has low sugar. Please refer to quotes number 22–23 in Supplementary Information 1.

### Physical capabilities

#### Physical skills

Such skills include the selection of cheap and healthy foods, selection of small portions of sweets, cooking/preparing healthy food, not adding sugar to foods or drinks, and the ability to determine the sugar content on food labels. Please refer to quotes number 24–26 in Supplementary Information 1.

However, some of the participants stated that they add sugar in food. Please refer to quotes number 27 in Supplementary Information 1.

### Social opportunities

#### Social influence

Parents and friends offer both positive support for the reduction of sugar consumption and a possible negative influence. The participants indicated that parents, for example, can limit the purchase of sweets or sugary foods. Also, trusted members of the family or society can encourage individuals to consume less sugar. Please refer to quotes number 28–29 in Supplementary Information 1.

In contrast, family members and friends may encourage the purchase of sweets or unhealthy food. Also, individuals can be influenced by a friend’s health condition. Please refer to quotes number 30–31 in Supplementary Information 1.

### Physical opportunities

#### Environmental context and resources

The environmental context and resources have a major influence on the consumption of free sugar. The cost of food may promote the consumption of cheap high sugar content foods. Also, the participants stated that social media can promote healthy food choices. Please refer to quotes number 32–33 in Supplementary Information 1. Participants raised many concerns about the fact that environmental context and resources can increases the consumption of free sugar. Many perceived there to be limited access to healthy food outside the home particularly snack foods. Also, participants voiced concern that healthy foods are expensive, and many low-income families have limited money for food. Advertisements and promotions can also contribute to an increase in sugar consumption. Please refer to quotes number 34–36 in Supplementary Information 1.

### Reflective motivation

#### Social and professional role and identity

Social and professional identities including part experiences, can play an important role in influencing sugar intake. Please refer to quotes number 37–38 in Information 1.

However, some participants stated that identities inherited from their parents and culture can increase the consumption of sugar. Also, an individual who was a student or on low-income may buy cheaper unhealthy foods. Please refer to quotes number 39–40 in Supplementary Information 1.

#### Beliefs about capabilities

A belief in your own ability to cook food, that it is easy to identify the amount of sugar in foods, that one is able to quantify sugar intake, that one is able to know sugar content by labels, that sugar content can be found on packaging, and that one can calculate the amount of sugar consumed per day can contribute to the reduction of free sugar intake. Please refer to quotes number 41–42 in Supplementary Information 1.

In contrast, if the beliefs about individual capabilities are weak or the individual is less confident, these beliefs can easily prevent behaviour change to reduce sugar intake. The following are examples of beliefs that were mentioned by the participants during the interviews: the difficulty of controlling sugar consumption or reducing sugar intake, feeling unable to reduce sweet snacks, the ease of getting sweets, laziness, hard to know the sugar content of meals, difficult to control the influence of advertisements, difficult or impossible to read the labels, and unable to understand the labels. Please refer to quotes number 43–47 in Supplementary Information 1.

#### Beliefs about consequence

Examples of beliefs about the consequences of sugar intake that will facilitate the reduction in free sugar consumption include the beliefs that sweets have unhealthy consequences and that naturally occurring sugars in fruits and vegetables and cooking food have healthy consequences. Also, the participants believed that placing a picture of tooth decay on food packaging would help them to reduce sugar intake and ultimately would prevent the need for dental treatment. Please refer to quotes number 48–50 in Supplementary Information 1.

In contrast, the following examples of such beliefs were expressed by the participants act as a barrier to reduce free sugar intake: the belief that exercise alone is sufficient to control weight without a reduction in food intake, the belief that performing exercise to burn energy means that the individual can eat sugary snacks, the belief that fizzy drinks boost energy. In addition, the selection of food with low calorie content can easily prevent behaviour change to reduce sugar intake. Please refer to quotes number 51–53 in Supplementary Information 1.

#### Intentions and goals

Examples of intentions that were expressed by the participants include the intention not to buy sugary foods, to reduce sugar intake; to eat healthy food, and to exercise. The participants indicated that their goals were to improve their fitness level and to reduce body weight. Please refer to quotes number 54–56 in Supplementary Information 1. None of the participants stated that they had an intention or aim to maintain their teeth by reducing sugar intake. Please refer to quotes number 57–58 in Supplementary Information 1.

### Automatic motivation

#### Social and professional role and identity

Social and professional roles and identities may influence free sugar intake unconsciously through automatic processes and choices. Routine cooking habits and parents’ modelling of cooking meals consisting of low-sugar foods can promote the reduction of free sugar intake to <5% of the total energy intake. Please refer to quotes number 59–60 in Supplementary Information 1.

However, habits can act as barriers toward reduce for sugar intake. For instance, the habit of sharing food with others and annual holidays can also increase the risk of consuming more sugar. Please refer to quotes number 61–62 in Supplementary Information 1.

#### Reinforcement

Reinforcement is thought to have a crucial role in facilitating reduced sugar intake. In the context of this study, reinforcement was associated with interventions as a facilitator of reduced free sugar consumption. Colour-coded labelling reinforces the selection of healthy food. Another example of positive reinforcement is the perceived reward of burning off energy through exercise and education about the effects of sugar reinforcing decisions about healthy food selection. Please refer to quotes number 64–65 in Supplementary Information 1.

#### Emotions

Participants reported both negative and positive emotions associated with the consumption of sweet foods, for example feelings of guilt after eating sweets, enjoying the taste of fruits and drinks without artificial sugar, love of having fruits with a main meal or as a snack, and dislike of sugary foods. Please refer to quotes number 66–67 in Supplementary Information 1.

In contrast, emotions can also drive individuals towards increased sugar consumption, serving as a strong barrier towards behaviour change. The following are examples of emotions that can prevent reduction of sugar intake to <5% of the total energy intake: cravings for fizzy drinks and sweets, love of Coca-Cola, enjoyment of tasty foods, consideration of sweets as treats, the love of a bargain, and no interest in counting sugars in foods. Please refer to quotes number 68–71 in Supplementary Information 1.

In Summary, 12 TDF elements are identified as being relevant to reducing free sugar intake to <5% of the total energy: Knowledge; Psychological Skills; Memory, Attention, and Decision Processes; Behavioural Regulation; Physical Skills; Social influence; Environmental context and resources; Social and professional role and identity; Beliefs about Capabilities; Beliefs about Consequence; Intentions and Goals; Reinforcement; and Emotions. Six of the COM-B Model elements which are relevant to reducing free sugar intake to <5% of the total energy: Psychological capabilities Physical capabilities Social Opportunities Physical Opportunities; Reflective Motivation; and Automatic Motivation.

## Discussion

The aim of this study was to explore the barriers and enablers to behavioural change to reduce free sugar intake related to dental caries in a sample of UK adults who identify their ethnicity as White. The findings from the study indicated that the COM-B model and the TDF domains provided a comprehensive framework for the description of the facilitators of and barriers to behaviour change to reducing sugar intake. This has been reported by other studies in different fields.^38–40^ This comprehensiveness of the COM-B model and the TDF domains has enabled the research to capture more factors related to free sugar intake than have been reported by previous studies.^16–25^ The factors are related to the following domains of TDF: Knowledge; Psychological Skills; Memory, Attention, and Decision Processes; Behavioural Regulation; Physical Skills; Social influence; Environmental context and resources; Social and professional role and identity; Beliefs about Capabilities; Beliefs about Consequence; Intentions and Goals; Reinforcement; and Emotions. From all 14 domains of TDF, the only exception to the framework, was that none of the participants’ comments could be mapped to the Optimism theme of Motivation. Altogether, these findings clearly indicated that the six elements of the COM-B model and the 14 domains of the TDF are relevant to behaviour change to reduce sugar intake.

In this study there were some limitations. one of the anticipated limitations in the study was the possible recruitment of participants from a middle-class academic setting only and that these participants would have little or no caries. Such a population would not reflect the full social range of the White ethnic groups in the UK, though some of the student participants reported coming from the lower social classes. Another limitation was that the authors were unable to assess the sugar content of the participants’ daily meals, as the brands and types of food were not verified in most of the cases. Despite these limitations, we conclude that this study highlighted many crucial factors that influence sugar intake in this population.

## Conclusion

The COM-B model and TDF framework provided an inclusive account of the barriers and facilitators of reducing sugar intake among white ethnic groups. Although the study had some limitations, we believe that the COM-B model and TDF can be useful for the development of strategies to address specific facilitators and barriers of the implementation of interventions that aim to reduce sugar intake to <5% of the total energy intake in relation to dental caries.

## Figures and Tables

**Table 1:** Characteristics of participants

*Characteristics*	*Frequency*
*Gender*	
Female	16
Male	11
	
*Job title*	
Staff	14
Student	13
	
*White Ethnicity*	
Scottish	1
English	10
British	7
Irish	1
Others	8
Age	Ranged from 19 to 59 years old
