# Supplementary material for: Factors related to reducing free sugar intake among white ethnic adults in the UK: a qualitative study
Source: BDJ Open. 2018 Feb 9;4:17024–. doi: 10.1038/bdjopen.2017.24 (PMC5842859; doi:10.1038/bdjopen.2017.24)
Supplement: Supplementary Information 1 [file bdjopen201724-s1.docx]

Supplement one:

| Domain | Influential | Quotes |
| --- | --- | --- |
| Knowledge | Facilitator | *1)"bad for your teeth aim biscuits sweets I think from what I was told is more about when you eat than what you eat, so if you eating between meal and the stuff is on your teeth all you day that's worse but also acidic food and sugar specifically."" (Participant-WT24)*  *2)" I think carbonate drinks are bad for your teeth. that what I can think of my top of my head "(Participant-WT25)* |
|  | Barrier | *3)"I have no knowledge of what the term free sugar means." " Yeah I don’t have any educational about sugar" (participant-W3)*  *4)“Like if its says 10% of sugar than I don’t know how much sugar is low and much sugar is high than I don’t know what that 10 % is mean.” (Participant-WT1).* |
| Psychological Skills | Facilitator | *5)"Well I know that for example the cereal that I have is 14% I think of my allowance for calories intake I know that banana is , I don’t know what banana is , but I know the salad is very low percentage so the turkey that I put in my salad is about 25% the salad is about 10% so with my two meals and my snack, my breakfast my snack and my lunch I now about 50% so that leave me 50% for my evening meal or anything else so that how I kind rationale things." (Participant-WT23)*  *6) "yes a lot of food I buy that healthy food tend to show the amount of the correct kind of sugar kind of in them, they will say let us assume so on the package they will say per 100 grams you know percentages and amount of sugar so I know if it has 30 grams of normal sugar and then 90 grams total of carbs per day and then best have what else say 20 grams saturated-fat 70 grams of normal fat in a day so I look in the package and normally have the values so you know calories carbohydrate of which sugars fats and salt....." (Participants-WT27)* |
|  | Barrier | *7) " aa so don’t do it in meals I don’t look at all really, in  may be snacks  I will look for calories and I will think of how many calories in in foods  and line meals   and kind of snacks things , but I wouldn’t often look how many grams of sugar."(Participant-W13)* |
| *Memory, Attention, and Decision Processes* | Facilitator | *8)"There is a brand very good but very naughty very bad for your chocolate called Vego its delicious it is 900 calories"(Participant-WT15).*  *9)"Drink every single drink I think is bad for you a lot of sugar, compare with water except for water and you know that’s it. and also you feel hard done by because the water is cheaper than the soft drink or the fruit juices " (participant-WT27)*  *10)"if I had the choice between like dried fruit that has that uses sugar as well that says that I don’t know apricot sugar and somethings I wouldn’t buy that, I would always buy the natural things" (participant-WT9)* |
|  | Barrier | *11)" I never think about the sugar intake." (Participant-WT3).*  *12)“ IT: alright, How easy do you find it to identify how much sugar is in your food or meals? WT18: I don’t pay attention to be honest to you I mean as far as I guess Intuitively I try to get every now may be this is foolish Intuitively I try and gauge whether you know if I am eating a farm loaded sugar can you know that clearly the ideal."(Participant-WT18).*  *13) " So I don’t think if I eat chocolate bar I think more about weight gain than my teeth definitely." (Participant-WT13).*  *14)"I love bargain I love bargain and if something on sale I would be more like to pay attention." (Participant-WT15)*  *15)" I don’t always necessary look at the kind of nutrient value I guess of a product if its half price." (Participant-WT23)*  *16) “I would not compromise for the taste. so if I went for the low fat and low sugar option and it didn’t taste good, I wouldn’t buy it again. “ (Participant-WT4).*  *17)“I don’t really trust most of packaging and labelling things a lot of it not really informative to the lay person.” (Participant-WT4)*  *18) " I think from what I was told is more about when you eat than what you eat, so if you eating between meal and the stuff is on your teeth all you day that's worse ." (Participant-WT24)*  *19)" I would say I usually go for , its healthy, there is usually one under 500 calories and one is over, so on the days that they have one under 500 calories I really like the look of it I will have that because its the healthy option again if there is something I don’t like either of the options I will go for jack of potato, just because again I don’t really like it, but its healthy and I don’t like the other two things okay they have." (Participant-WT14)* |
| *Behavioural Regulation* | Facilitator | *20) “So I plan the entire day around this so as I said I wake as I said I need some sugar today I am going to have may be yogurt with some fruits in the morning and have a porridge may be slightly later and then have big lunch then after lunch have couple of coffee with milk and then you know I am like energized and full of energy when I get there because I have sugar still in there and my lunch still working for me so when I go to the gym a I burning off complete like in zero and I tend not to eat for about half an hour after I leave the gym because when I get home I get my dinner so I have the meat with vegetables, and that just." (Participant-WT20)*  *21)"Yeah, I would, if I was to buy the big bars do you know like one pound for Cadburys big bar I will just eat it all, so just buy the 60 pens little small bars and I would have a bar of that.” (Participant-WT7)* |
|  | Barrier | *22)"I think I would first think about kind of put my fitness and weight before I think about teeth." (Participant-WT1).*  *23)"yeah, I think I mainly look at calories and then if its got one of those you know there some package have some five little boxes where has like fat sugar and salt and something else and they have got different like red yellow and green, the more green I get the better " (Participant-WT14)* |
| *Physical Skills* | Facilitator | *24)" She wants me to help her because I am quite efficient because I find real cheap and healthy food and get them but she is quite bad for it."(Participant-WT27)*  *25) "Drinks teas and coffees are the things I drinks the most of, I drink them at work during the day, I don’t add any sugar on" (Participant-WT15)*  *26)"if I look in the labels that I can find some kind of sugar content" (Participant-WT18)* |
|  | Barrier | *27) ” I do put  sugar in it,  for some strange reason I can only I like tea but it has to have some sugar in it,  like half spoon still I want to have sugar in there because of the flavor  of tea on its own I don’t really like unless it’s a bit  sweet where its coffee its a bit different.****(Participant-W7)*** |
| *Social influence* | Facilitator | 28)*“My mum does sort of put a limits, on she like you can’t buy too much of like you know sweets or you know you’ve got make sure you try to keep it quite balanced so you know you must have this amount of fruits this amount of she there is more concern that like the main meal is like what I am focusing on the bigger part and what I am buying as opposed to like any sweets or. Yeah.”(Participant-WT22).*  *29) "My interest in Japanese street fashion does affect my choices when buying food because I try to eat healthy so I can hopefully fit the small sizes better "(Participants-WT1)* |
|  | Barrier | *30) "My other daughter like biscuits like ready made biscuits of like digestive she like that those I will be them for her aam, yes the list is made buy the girls and dad goes.... as I said before that my eating habit tend to be shared so when the biscuits gets opened I am in there too [laughing] " (Participant-WT17). 31)"Occasionally we go out for dinner with my friends and my partners that less healthy so might have a burger with chips." (Participant-WT3).* |
| *Environmental context and resources* | Facilitator | *32)" For example they have a lot of vegetable which one I think its a 60 p value and then like right at the from of the store as soon as you go in my store anyway so that influence me to choose that vegetables." (Participant-WT7).*  *33)"Yeah and fruits is free at university so we can take it." (Participant-WT16).* |
|  | Barrier | *34)"I think if I wasn’t too worry about the money at that time I would go for a healthier option.""(Participant-WT1) 35)"Yeah so sometime when I am sick now when I am adult I buy the Lucozade."(Participant-WT25).*  *36)“Now money is more of a factor I have moved I don’t have easy access"(Participant-WT18).* |
| *Social and professional role and identity* | Facilitator | *37)" I think it will influence me to change what I eat like I had flu a couple of months ago and I completely lost my appetite and that was a change for short but then when I start to get again I was really careful what I eat I was really trying to eat healthy food am." (Participants-WT14) 38)" I did go though phases ages ago trying to cut down sugar and I was amazed how much stuff has so much sugar in it like a bottle of orange juice has like a 50% of sugar intake or something I was just I said its impossible. [Laughing] so" (Participant-WT14)* |
|  | Barrier | *39)"aa yeah , as a student you trying to get the cheapest you can yeah. " (Participants-WT16)*  *40) "I think if the food is more expensive than families with low income for example like me or students budget aam wouldn’t buy sugary things even though I don’t buy that much sugar "(Participants-WT7)* |
| *Beliefs about Capabilities* | Facilitator | *41) "no its I would be you know even think of cooking dinner I have my three potatoes and my one pepper and yes I can find the sugar contents of all of these things but I would have to then calculate how much it added up to for example I think for orange juice I have in the morning I can work it out quite easily because what tells me in the pack." (participant WT25)*  *42)"you know I do like my sweety things I try I restricted myself to have pudding rather than snacks whole day and I know a lot of friends who eat a lot of sugar so I guess that how I can quantify it up for myself."(Participants-WT12)* |
|  | Barrier | *43)"I have always I think it might be because I would still find it real hard to give it up like I was having a craving for it still and I thought this is an excuse to given to the craving. yeah and then I just got back to the habit of it and I would like to stop drinking it again." (Participants-WT14). 44)"the truth is even having read about it I don’t’ understand that much about so I would rarely look at the labels" (Participants-WT24)*  *45)" probably laziness [laughing]. I just yeah I just don’t, I think if I care more by myself and what I was eating then I would probably think about it and I again I do go through phases where I follow a diet plan or something like that but again its rare , day to day i don’t care enough I suppose."(Participants-WT10)*  *46)"so I think I have been exposed to so much television and mainly television but also kind of billboard advertisement for fizzy drinks that its almost an subconscious kind of i don’t have control over it any more so or that much control so if hear someone open a can of coco I kind I want a can of coco or if i seen someone drink a can of coco I kind want on" (Participants-WT25).*  *47)"when I shop if I could see a clear label stating how much sugar its actually in the product but yeah its not that easy to read at the moment... its not easy now because you really have to look for it you kind you have to analyse it so." (Participants-WT20)* |
| *Beliefs about Consequence* | Facilitator | *48)"chocolate and chocolate bars and things like that normally is artificial sugar that’s’ not very healthy for you so accept. and the good sugar fructose and all the you know natural occurring sugar is in fruits and vegetable then to be perceived as more healthy so yeah my understanding of sugar is definitely."(Participants-WT20)*  *49)" yeah I think if aam on a day when I done exercise aam it can go either way, either if you feel good about having exercise and so more healthy and avoid snack ....."(Participants-WT11)*  *50)"I think tooth decay image would be the best because if someone can see it then they you know attract the eyes a bit more as opposed to number or colours I don’t think. in the way that it will scare me in to thinking that you know me teeth will goanna rot way or you know , i want try to avoid as much as possible if I can you know getting replacement teeth for denture when I get older or anything like that".(Participants-WT22)* |
|  | Barrier | *51)"I will have fizzy drinks if I am sleepy may be and just want a little boost" "I think but I know that in moderation these products that high sugar is fine already I belief its fine and I belief that I am using in moderation" (Participants-WT25)*  *52)"I think if its taste, will put it this way I don’t see any point in having aam say low fat low sugar yogurt if it doesn’t taste anything” (Participant-WT26)*  *53)"aam amm i would say i usually go for , its healthy, there is usually one under 500 calories and one is over, so on the days that they have one under 500 calories i really like the look of it I will have that because its the healthy option agin if there is someting i don’t like either of the options I will go for jack of potato, just because again i don’t really like it, but its healthy and i don’t like the other two things okay they have." (Participant-WT14)* |
| *Intentions and Goals* | Facilitator | *54)"i want to get my physical aa my physic in shape so the food i buy you know i am not going to buy ice cream or crisps " (Participants-WT3)*  *55) " I kind of being in my idea of you know cutting down sugar and eating healthy diet I just wanted to try you know this meal" (Participants-WT20)*  *56)“I want to loss weight (Participants-WT17)* |
|  | Barrier | *57)“Again if you decided to go and buy lunch I have already have decided I already know its not be going to a healthy choice I won’t really consider it."(Participants-WT25)*  *58)"aam so because like I play in full as well the aim is to gain weight"(Participants-WT27)* |
| *Social and Professional Role and Identity* | Facilitator | *59)"I think in my idea and how I was brought up sandwiches in the morning are not suppose to be too sweet. " (Participants-WT20)*  *60)" Generally speaking I am creature of habit when it come to my shopping I try to buy I again it does depend on the food stuff I try to buy as far as fruits and veges" (Participants-WT18)* |
|  | Barrier | *61)"I had I started drinking them again when I went on holidays because I thought I am on holiday so it’s fine [Laughing] yeah and got back and I said well it’s still kind of like holiday feelings so I just have may be one and then aam yeah just got back into the habit."(Participants-WT14)*  *62)"I said before that my eating habit tend to be shared so when the biscuits gets opened I am in there too [laughing]"(Participants-WT17)* |
| *Reinforcement* | Facilitator | *63)"The traffic light it shows you know its gives you positive reinforcement if you get something which is all green you got some rice cake or whatever than or anything fill with some harm or chicken or whatever you what" (Participants-WT27)* *64) "IT: amount of exercise to burn of energy, so if you have a product and they say if you consume this product you would need to run for half of hour to burn your calories , do you think this will help? WT11: yeah I think that’s quite a good good incentives particularly aam for someone my position who exercises some what but not a lot." (Participants-WT11)*  *65) "IT: okay , education about sugar in food , do you think education about sugar in food would help you to select lower sugar lower foods, foods with lower sugar? WT25: yeah probably I am not sure how much of influence it will have , it probably reinforce the decision I am making already or might help to make better once yes I think it will have a positive effect." (Participants-WT25)* |
| Emotions | Facilitator | *66)"yeah I love to have my fruits with my lunch or as a snack I would have it at about in 3 pm."(Participants-WT25)*  *67) "aam I don’t ever buy fizzy drink I don’t like them especially aam I don’t like coco the one which is not diet because it leave like often with a lot of fizzy drinks it leaves like a film of roughness on your teeth so don’t drink fizzy drink for that reason" (Participants-WT7)* |
|  | Barrier | *68)" I love coca cola as it is not coca cola zero because for me it’s different taste than I will not go for coca cola zero, just because for me it’s a different taste."(Participants-WT6)*  *69)" if I am going out for a meal I just want something really tasty because its a treat you don’t do it very often do you."(Participants-WT10)*  *70)"I love bargain I love bargain and if something on sale I would be more like to pay attention." (Participants-WT15)*  *71) "I have no motivation or interest in calculating sugar content of my food that I prepare myself because I don’t consider to be higher in sugar. "(Participants-WT25)* |
