# Supplementary material for: Factors related to reducing free sugar intake among white ethnic adults in the UK: a qualitative study
Source: BDJ Open. 2018 Feb 9;4:17024–. doi: 10.1038/bdjopen.2017.24 (PMC5842859; doi:10.1038/bdjopen.2017.24)
Supplement: Supplementary Information 2 [file bdjopen201724-s2.docx]

Supplement Two:

| Theme | Subtheme | Related Quotation |
| --- | --- | --- |
| Knowledge | Knowledge of food and drinks cause dental caries | "Bad for your teeth biscuits sweets …. also acidic food and sugar specifically." (Participant-WT24)  "so I think, fruits with high sugar content aam sweets with high in sugar content also I think carbonate drinks are bad for your teeth aam. that what I can think of my top of my head " (Participant-WT25) |
|  | Knowledge of healthy foods for teeth | "IT: What food do you consider are healthy for your teeth? WT2: Healthy vegetables fish" (Participant-WT2) |
|  | Knowledge of acidic food | "...also acidic food and sugar specifically." (Participant-WT24) |
|  | Knowledge of the amount of sugar in food | " Yeah I know I have no idea how much sugar is in fruits I couldn’t even has it a guess I know there is natural sugar in fruits, but I don’t how much of these is bad for you or not."(Participant-WT14)   "my food that I prepare myself because I don’t consider to be higher in sugar. " (Participant-WT25) |
|  |  |  |
|  | Knowledge of the time for sugary food | "so I think, fruits with high sugar content aam sweets with high in sugar content also I think carbonate drinks are bad for your teeth aam. that what I can think of my top of my head . that what things I associated with and I am careful when I am consuming and I consider them bad for my teeth." (Participant-WT25) |
|  | Knowledge of the term Free Sugar | "free sugar I wouldn’t know aam [laughing] I don’t know free flowing sugar like from a bag, I am not sure to be honest I have never heard that term before. " (Participant-W10) |
|  | Knowledge of the term Added Sugar | awareness of the term Added sugar was limited to 7 out of 27" |
|  | Knowledge of recommended daily intake | " Like if it’s says 10% of sugar than I don’t know how much sugar is low and much sugar is high than I don’t know what that 10 % is mean. I need to know okay below 10% is good and above 10% is bad something like that. If I know that then I can judge what that number means. but if you just give me a number and I don’t know what does mean, then I can’t use that in my decision."(participant WT1). "my food that I prepare myself because I don’t consider to be higher in sugar. " (Participant-WT25) |
|  | Knowledge of colour coding system | " thinking about now I don’t actually know what green means I just know that means good but I don’t know what the cut off is for each one." (Participants-WT14) |
|  | Knowledge about food calories | "aam amm I would say I usually go for , its healthy, there is usually one under 500 calories and one is over, so on the days that they have one under 500 calories I really like the look of it I will have that because it’s the healthy option again if there is something I don’t like either of the options I will go for jack of potato, just because again I don’t really like it, but it’s healthy and I don’t like the other two things okay they have." (Participant-WT14) |
|  | Knowledge of food labelling system | "from what I have read it, it’s a complicated thing you know how they put it and then the difference in different kind of sugars, the truth is even having read about it I don’t’ understand that much about so I would rarely look at the labels." (participant WT24). |
|  | Need for knowledge on effect of sugar on the body | "I think you know if you show some fact like actual facts that goes to body you know like body like VC you know if you compare the fats and muscles and grams because I see stuff like in the internet as well may be that could help I don’t know " (participant WT20). |
|  | Need for knowledge on effect of sugar on the teeth | " aam I would say there could be more of information about natural sugar and dangerous they could pose to someone’s teeth and the health of their teeth" (participant WT23) "I think you know if you show some fact like actual facts that goes to body you know like body like VC you know if you compare the fats and muscles and grams because I see stuff like in the internet as well may be that could help I don’t know " (participant WT20). |
|  | Need for knowledge on the recommended daily sugar intake. | " I would like to have information that’s in context and gives me a better idea of the levels of sugar that not merely in individual foods but that is reasonable so if you are talking about under 5% what does it mean on ordinary diet what would that actually mean in terms okay mean someone like me I don’t know sugar in my tea and coffee so the sugar that I see going into my food is if my wife makes a cake I know that she puts that amount of sugar into the cake, then I have a piece of cake so its percentage of that sugar, but a part from that I don’t really know what’s, do you understand what I am saying, so telling me a percentage that I need or shouldn’t exceed is one thing but actually what’s really means in terms of day to day of normal food that’s I would like to be educated on better so I can make better choices based on that." (participant WT17) " I need to know okay below 10% is good and above 10% is bad something like that. If I know that then I can judge what that number means. but if you just give me a number and I don’t know what does mean, then I can’t use that in my decision."(participant WT1). |
|  | Need for knowledge on colouring coding system. | " I do like to read labels I do like to understand how this is it’s a red label and its say 45 % 45% of what ? and then its say its daily intake" (Participants-WT20) |
|  | Need for knowledge on hidden sugars. | "think it’s very important to educate people on the hidden content of foods if you know what I mean " (participant WT7) |
|  | Need to understand the application of the recommended daily sugar intake in everyday life | "I would like to have information that’s in context and gives me a better idea of the levels of sugar that not merely in individual foods but that is reasonable so if you are talking about under 5% what does it mean on ordinary diet what would that actually mean in terms okay mean someone like me I don’t know sugar in my tea and coffee so the sugar that I see going into my food is if my wife makes a cake I know that she puts that amount of sugar into the cake, then I have a piece of cake so its percentage of that sugar, but a part from that I don’t really know what’s, do you understand what I am saying, so telling me a percentage that I need or shouldn’t exceed is one thing but actually what’s really means in terms of day to day of normal food that’s I would like to be educated on better so I can make better choices based on that." (participant WT17) |
|  | Need for knowledge on healthy eating | " yes, I think would aam I think especially for me realise the different between or perhaps need information about the difference between the different types of sugars amm but then you get all education knowledge about what you are eating and how it all what’s there I think is all in the sugar it is useful." (Participants-WT26) |
|  | Need for knowledge on different types of sugar. |  |
|  | Need for knowledge on sugar content of processed foods and ready meals. | " I think it’s important that people know exactly what is good and what is bad especially when it’s come to the processed food and ready meals because people think that for example a ready meal Bolognese wouldn’t have any added sugar on it because it’s something you would not think it’s sugary when it’s have a lot of sugar in it make the flavour’s right for example." (participant WT7) |
|  |  |  |
|  | Need for knowledge on effect of exercise on sugar intake. | "I am not so sure that would help half an hour of what ? half an hour of high intensity exercise or just half an hour of exercise in general I think that would wind up in a way I am suspect may be more information than you could easy on a label or product without you know minimizing it to a point it doesn’t mean anything anymore. "(participant WT17) |
| Psychological Skills | Ability to calculate daily intake of sugar | "aam well I know that for example the cereal that I have is 14% I think of me allowance for calories intake I know that banana is , I don’t know what banana is , but I know the salad is very low percentage so the turkey that I put in me salad is about 25% the salad is about 10% so with me two meals and me snack, me breakfast me snack and me lunch I now about 50% so that leave me 50% for evening meal or anything else so that how I kind rationale things." (Participant-WT23) |
|  | Ability to assess sugar content in foods and meals | "so the most commonly used one is the circle with different segments in it you know the one I mean and I think some understanding of sugar how much sugar is in something compared to the recommended limits so you can see how much this is given you toward overall recommended limit, which help inform you know am I going to use all of me sugar allowance on this one thing because I really wanted whereas you know if I have this other things it’s only got half the amount so that leave another half for something else later on the day. "(Participant-WT19) |
|  | Ability to interpret food labelling | "yes a lot of food I buy that healthy food tend to show the amount of the correct kind of sugar kind of in them, they will say let us assume so on the package they will say per 100 grams you know percentages and amount of sugar so I know if it has 30 grams of normal sugar and then 90 grams total of carbs per day and then best have what else say 20 grams saturated-fat 70 grams of normal fat in a day so I look in the package and normally have the values so you know calories carbohydrate of which sugars fats and salt....." (Participants-WT27) |
| Memory, Attention and Decision Processes | Prioritizing frequency of eating sugar over the amount of sugar | " I think from what I was told is more about when you eat than what you eat, so if you eating between meal and the stuff is on your teeth all you day that's ." (Participant-WT24) |
|  | Choose food with lower sugar | "Drink every single drink I think is bad for you a lot of sugar, compare with water except for water and you know that’s it. and also you feel hard done by because the water is cheaper than the soft drink or the fruit juices " (participant-WT27) "if I had the choice between like dried fruit that has that uses sugar as well that says that I don’t know apricot sugar and somethings I wouldn’t buy that, I would always buy the natural things" (participant-WT9) |
|  |  |  |
|  | Trust of food labelling | “I don’t really trust most of packaging and labelling things a lot of it not really informative to the lay person.” (Participant-WT4) |
|  | Remember /Retain advice on sugar intake | " a friend who is a dentist and she told me not to put sugar anymore, so about 5 years I got stopped putting sugar."(Participant-WT5). |
|  | Remember/Retain information of sugar content in foods | " there is a brand very good but very naughty very bad for your chocolate amm called Vega its delicious it is 900 calories"(Participant-WT15). |
|  | Main attention is on food calorie | "yeah, I think I mainly look at calories and then if it’s got one of those aam you know there some package have some five little boxes where has like fat sugar and salt and something else " (Participant-WT14) |
|  | Choose food with low calories | "aam amm I would say I usually go for , its healthy, there is usually one under 500 calories and one is over, so on the days that they have one under 500 calories I really like the look of it I will have that because it’s the healthy option again if there is something I don’t like either of the options I will go for jack of potato, just because again I don’t really like it, but it’s healthy and I don’t like the other two things okay they have." (Participant-WT14) |
|  | colour coding help on decision making on food selection | " a traffic light system so like the green the orange and red I think they are useful just at glance specially when comparing two products.... just aam at a glance I think when you are in rash it might not be the most accurate means aam but again if you are comparing two products I think it’s very quick way of choice yeah."(Participant-WT11) |
|  | Colour coding helps attention | "they have it’s both on the traffic light thing so it has the little label it has the colour in which attract your eye." (Participant-WT7) |
|  | colour coding help on decision making on food selection | "Well I mean you know naturally looking something red it says you know around everywhere it says stop or warning or alert or something so if you see something is green on it I will draw your eyes first to second attention you will look at it and say oh its healthy its fine and it has some red on it you will know kind of you can compare things better if I am looking at two sets of numbers let say I don’t know chicken breast vs chicken thighs for example or different types of mincemeats if I am sticking at two set of numbers it’s hard to compare them among each other but if say one had all green and one had three green and two yellow then you could quite easily see that the green one is better and in one part so I think it’s very useful" (participants-WT27) |
|  | Pay attention to low price food | "I love bargain I love bargain and if something on sale I would be more like to pay attention." (Participant-WT15) " I don’t always necessary look at the kind of nutrient value I guess of a product aam if its half price." (Participant-WT23) |
|  | No Attention on sugar intake | " I never think about the sugar intake." (Participant-WT3). |
|  | Body health is more important than teeth health when consuming sugar | " so I don’t think if I eat chocolate bar I think more about weight gain than my teeth definitely." (Participant-WT13). |
|  | Prioritize food taste over health | "Although I know the risks of consuming a lot sugar still I do , despite that because I prioritizing taste as suppose over the health risk"(Participant-WT11)   “I would not compromise for the taste. so if I went for the low fat and low sugar option and it didn’t taste good, I wouldn’t buy it again. “(Participant-WT4). |
|  | Prioritize sugar intake when buying food over buying meals | "I wouldn’t be I mean I just assume as not as much sugar and its less important to me if the sugar in like curry but more impotent if I am having like a snack."(Participant-WT16) |
|  | No Attention on sugar content | " IT: alright, how easy do you find it to identify how much sugar is in your food or meals? WT18: I don’t pay attention to be honest to you I mean as far as aam I guess Intuitively I try to get every now may be this is foolish Intuitively I try and gauge whether you know if I am eating a farm loaded sugar can you know that clearly the ideal."(Participant-WT18). |
|  | No attention on Nutritional Values | "but I wouldn’t often look how many grams of sugar." (Participant-WT13) " I don’t always necessary look at the kind of nutrient value I guess of a product aam if it’s half price." (Participant-WT23) |
|  | Tooth decay image helps in paying attention | "I think tooth decay image would be the best because if someone can see it then they you know attract the eyes a bit more as opposed to number or colours I don’t think. ".(Participants-WT22) |
|  | Pay attention to calories and fat saturated fats | "I tend to focus on like the calories and fat saturated fats because I know that it’s bad for you" (Participant-WT14) |
|  | No attention on colour coding | "To be honest it would need to be quite well kind of publicised and advertised otherwise I would not think of colours."(participant-WT10). |
| Behavioural Regulation | Daily plan for sugar intake | "so I plan the entire day around this so as I said I wake as I said I need some sugar today I am going to have may be yogurt with some fruits in the morning and have a porridge may be slightly later and then have big lunch then after lunch have couple of coffee with milk and then you know I am like energised and full of energy when I get there because I have sugar still in there and my lunch still working for me so when I go to the gym a I burning off complete like in zero and I tend not to eat for about half an hour after I leave the gym because when I get home I get my dinner so I have there meat with vegetables, and that just."(Participant-WT20) |
|  | Selecting small size of sugary food | "yeah , I would, if I was to buy the big bars do you know like one pound for Cadburys big bar I will just eat it all, so just buy the 60 ps little small bars and I would have a bar of that." (Participant-WT7) |
|  | Prioritise fresh foods then carbs | " when I cook when I buying food obviously my priority fruits and vegan and then carbs " (Participant-WT12). |
|  | body weight comes first then oral health when eating | "I think I would first think about kind of put my fitness and weight before I think about teeth." (Participant-WT1). |
|  | Food calorie comes first then other elements of labelling | "yeah, I think I mainly look at calories and then if it’s got one of those aam you know there some package have some five little boxes where has like fat sugar and salt and something else and they have got different like red yellow and green, the more green I get the better " (Participant-WT14) |
|  | Need for self-monitoring system | " I don’t think so other than like I say I would find it genuinely useful to have some kind of way monitoring my own sugar intake that was Doable without you know I am not sure I really know how much sugar in my diet." (Participant-WT17) |
| Physical Skills | Select healthy and cheap food | " she wants me to help her because I am quite efficient because I find real cheap and healthy food and get them but she is quite bad for it."(Participant-WT27) |
|  |  |  |
|  | prepare healthy food for cooking | "when I cook I tend to cook something Japanese so we would have either kancho or we could have miso soup with some rice and vegetables we would have Japanese curry which is slightly heavier we would have for lunches aam so this is one big hobby influence me." (Participant-WT20). |
|  | find sugar content in food label | "if I look in the labels that I can find some kind of sugar content" (Participant-WT18) |
|  | Need for cooking skills | "also show them how to cook and not to rely on paying from the shops."(Participant-WT2) |
| Social influence | Parents Influence on sugar consumption and healthy eating | " my mum does sort of put a limits, on she like you can’t buy too much of like you know sweets or you know you’ve got make sure you try to keep it quite balanced so you know you must have this amount of fruits this amount of she there is more concern that like the main meal is like what I am focusing on the bigger part and what I am buying as opposed to like any sweets or. yeah ." (Participant-WT22). |
|  | Parents encourage sugar intake with exercise | " IT: you told me that you parents also told you that is fine if you are young to have sweets. WT16: yeah well in a way I mean if you going to burn it off then it’s okay."(Participants-WT16).  "aam not really, I tend to just eat lots of fruits which obviously sugar but it’s easy and you know when I was ill when I was younger my mum would always give a lot citric fruits because it has a lot of vitamin c that help dealing with flu."(Participants-WT20)  "I think in my idea and how I was brought up sandwiches in the morning are not supposed to be too sweet. " (Participants-WT20) " that I was what brought up eating it’s quite easy to cook you can make a lot on Sunday and last for the rest of the week you stick in the microwave [laughing] ." (Participants-WT19) |
|  | Parents' beliefs are a strong influence on my beliefs about sugar intake |  |
|  | Parents wouldn't like children to buy sweets with a picture of tooth decay | " yeah probably I don’t know I just can’t see it happening just because like kids like package of skittles whatever I don’t think the parents which really like it if their kids come home with package of skittles with wiki teeth on them"(Paerticipant-WT14) |
|  | Partner Influences on eating | "yeah , probably if she comes with me she might say let’s try this or try that ...... yeah , I am quite flexible , I think." (Participant-WT16). |
|  | Friends Influences on eating | "occasionally we go out for dinner with my friends and my partners that less healthy so might have a burger with chips." (Participant-WT3). |
|  | Culture Influences on eating | "my interest in Japanese street fashion does affect my choices when buying food because I try to eat healthy so I can hopefully fit the small sizes better aaaa"(Participants-WT1) |
|  | Health professionals influence on eating | "yeah I suppose I might be effected by people whose judges I trust for example if my brother came to my he is a doctor and said to me , for example I buy we have long stand we talk about things occasionally food related , and he is of the opinion you should not have margarine you should have a butter because he think that margarine is so processed is much worse for you than any other stuff that’s native aspect of butter. So for example I have charted with him about that and now I buy butter rather than margarine, so he affect me and if he was to say to me don’t buy the Lloyd Grossman pasta sauce because it’s so processed it’s got so much sugar you know it’s really any it’s going be bad for you in long run, then I would listen to him. " (Participant-WT-18) |
|  | Friends' experience of ill health influence on eating | "like I have friends how won’t drink fizzy drinks and stuff because she’s have so many fillings but I have had one filling about 6 years ago , so I don’t think if I eat chocolate bar I think more about weight gain than my teeth definitely."(Participant-WT13). |
| Environmental context and resources | Restaurants foods are expensive | " aam well for lunch especially I have home made because of price first of all can’t afford to go to Pret every day to get a to get a sandwich and then I choose it also on what something what’s going to be filling because I get hungry a quite a lot and then taste as well am and also health but I consider health in all my meals not just I am making that food to be healthy if I wasn’t make that food than I would still choose a healthy options if I have enough money to go." (Participant-WT7). |
|  | Healthy food are cheap | " for example they have a lot of vegetable which one I think it’s a 60 p value and then like right at the form of the store as soon as you go in my store anyway so that influence me to choose that vegetables." (Participant-WT7). |
|  | Free fruits in work place | "yeah and fruits is free at university so we can take it ." (Participant-WT16). |
|  | Social media is a source of information for foods and health | " aaam probably little a bit I have actually seen some of advertise they are not advertisement but you know like you say in the Facebook like a little thing you can click on it and you can see what you want to buy, I click on one of them and told me a bar of chocolate is this many setups or a bar or bowl of pasta is this long in the gym , do you know that kind of thing from actually seeing that" (participant WT7) |
|  | Drink more sweets drinks when ill | "yeah so sometime when I am sick now when I am adult I buy the Lucozade."(Participant-WT25). |
|  | Drink more fruit juice when ill | " aam so if I ever get if start get cold or feel a bit ill I will always drink a lot of apple juice or eat some oranges juice or take some vitamins or something like that, because I think it might help but I might be too late and then I have to get." (Participant-WT13). |
|  | Eat more Sweet when ill | "Yes I have actually I have just been sick and If I have to go shopping when I am sick amm it’s harder for me to kind of resist the unhealthy food. Like normally I have some well power like now I shouldn’t do that that’s not good for me [smiling], but when I am sick that doesn’t work I just go for whatever easy and tasty because usually if I am sick I don’t really feel like eating so I need to make something very appetising for me to make sure I still eat enough , so I eat less healthy when I am sick [laughing] usually."  (Participant-WT1). |
|  | Limited access to healthy food. | " Now money is more of a factor I have moved I don’t have easy access" (Participant-WT18). |
|  | Store promotions (offers half prize influence food selection | " yeah I think I am definitely influenced by promotion so if something has the coloured labels or has a half price sticker or buy one and get one free something like that I do get drown by those things" (Participant-WT23). "they have been so many year bla bla ba so to buy their , the whole box of 30, they were like £ 50 now I understand your thinking £ 50 who spends a £ 50 on a box of chocolate , but I was like waw this is a really good deal and when we buy them they normally 3.58 £ now they working out less then £2 each I will buy them""(Participants-WT15) |
|  | Advertisements Influence food selection | " I think I am a victim of advertisement"(Participant-WT25) |
|  | Accessibility of sweet foods | "so it depends on what I am doing if I am am if I am at home I have a lot of snacks like biscuits and chocolates and rubbishy stuff if I am not at home than I don’t tend to have that stuff so much I might have if I am not at home during the day I might snack once with something like that biscuits or chocolate bar am if I am at home all day than it’s probably three times in the day I will snack." (Participant-WT11) "there is [laughing] a lot of snacks in our office and it’s very hard to avoid them , aam probably biscuits mot very many."(Participant-WT19) |
|  |  |  |
|  | Limited budget influence food selection | "I think if I wasn’t too worry about the money at that time I would go for a healthier option."(Participant-WT1)  " Now money is more of a factor I have moved " (Participant-WT18). |
|  | Difficult to know sugar content of restaurant meals | "aaam I mean I rarely I have takeaways but with takeaways I find it impossible to know how much sugar that was" (Participant-WT19) |
|  | TV programmes or advertisements influence choice of eating | "you know quite a lot of people are influence by TV programme, or government are influenced by researcher who may be lopping behave the industry rather than you know for the good of the population"(Participant-WT19) |
|  | Many foods and drinks have too much of sugar | " I think there are too many foods and drinks over filled with sugar because it make them more palatable and cheap and it will be for example I do try and avoid things which fruit taste syrup and things like that’s it seems just cheap white and palatable I would rather not have that kind of stuff."(Participants-WT17) |
|  | Sugar contents are on the packaging of foods | "yeah, I think I mainly look at calories and then if it’s got one of those aam you know there some package have some five little boxes where has like fat sugar and salt and something else " (Participant-WT14) |
|  | Labelling is difficult to understand | "from what I have read it, it’s a complicated thing you know how they put it and then the difference in different kind of sugars, the truth is even having read about it I don’t’ understand that much about so I would rarely look at the labels." (participant WT24). |
|  | Size of a serving on food labelling is difficult to understand | "what isn’t clear when aam if you not sure what way to use something you are using so for example like if they give sugar content per 100 grams and then per serving I think it’s not always clear how much sugar in each serving because you don’t know what size the serving is if that make sense aam so if you buy chocolate bar sometime a serving is actually is only a half of that so if you just glance at it then you will be like ooh that’s okay. but actually if you eat the whole thing then which you would because they are small that for one person [laughing] I think".(participant WT11) |
|  | Food products have colour coding system | " they have got different like red yellow and green, " (Participant-WT14) |
|  | Food labels are often in a small fonts | "it’s in teeny tiny in a font you can’t read so if it was clear I would be happier." (Participants-WT20) |
|  | TV shows educate about sugar | " I would say when I heard about Jamie Oliver doing his TED talks with abc in his TED talks for with big wheel barrel of sugar and the tips on the stage what I saw a tiny snip of that I what it to say the whole speech" (Participants-WT15) |
|  | Limited campaigns about reducing sugar consumption | "because there is no like you talking about government information knowledge you know we have a governments you know campaigns about smoking illness or alcohol abuse or eating too much fat but there is nothing telling me about sugar consumption. "(Participants-WT3) |
|  | Apps not helpful to reduce sugar intake | "aam it’s quite difficult I did try new for a while my fitness pal you know the app you put in your phone so that you can put and it’s supervise me while I thought I was being really kind of careful you know when you do my fitness pal you are trying to be healthy aam I got to the point where I was like I am actually going to ignore it what it tells me about sugar because it just went it was away over for me you know I cancel the fruits and things that counted towards I would know aam I could have as many bananas [laughing] and apples that I could and it didn’t matter and may be only keep cut of the sugar and stay something like my muesli bar or my soya milk and things like that with added , I am how were my , is that the question how were ?" (Participant-WT15) |
|  | No apps count sugar content | " that good do yeah I mean I have an app in my phone that kind of does that ammi used for running but also allows you to enter the food that you eating and will break down and shows you that’s how much fat you had this is how much protein and this is how much carbs, no sugar on it just carbs fat and protein"(Participant-WT10) |
|  | Food labels need to be wider and big warning number | " to have wide label with black text and say this is the amount of sugar grams and have like to recommended daily intake for men and women and obviously just to make it easy to read if you if I pick something I can see clearly sugar that many grams percentage for women percentage for men okay yeah okay" (Participants-WT20) |
|  | Food labels need to be more clear |  |
|  | Food labels need to have Daily recommendation intake of sugar for both gender |  |
|  | Food labels need to be in percentage form | "I would prefer percentages but ...just because they seems easy to understand quickly whereas having to convert you know let’s say you are only allowed 10 grams and you used 3.5 grams in this I don’t know.(Participants-WT19) |
|  |  |  |
|  |  |  |
|  | Food labels needs to have recommended daily intake against percentage of sugar content. | " so I think the percentage of the content would be more like a point of interest GP like ooh okay whereas the percentage of your daily is more likely to inform you the choices so if you know that by eating this type well this 50% of something for day then you might think twice about it. this what I want my 50% to be or something else that I want more , aaam so I think the percentage of your daily would be the most useful if you trying to reduce your intake" (participant WT11). |
|  | Food labels need to have total amount of sugar of a product | " so they say they give you instead of like half bottle or third of bottle they actually tell you what’s in the whole of the bottle, do you know what I mean ?"(participant WT14). |
|  | Food labels state less than a total amount of sugar of a product |  |
|  | Food labels need to be in simple colour scheme | "I guess I mean it’s over simplifying where if you have something like said you know there like energy rating on your fridge where you have got 5 and colour coded, so I know some food have say for example, high amount of sugar and that would okay but if every food kind of have something regarding I don’t concentration of something so you know if you buy a jar of honey is like 100% am because it wouldn’t be a soly negative thing but it will let you make up your mind you know what in each thing you could eat it in in accordingly quantity you know."(participant WT24). |
|  | Food labels font need to be big and in front of products | " well I know there is ingredients it’s difficult but in the front either got big block and it says sugar fat and protein I mean I don’t know about the traffic light system but I think it in the front and it’s said there is X amount of grams of sugar in this.... I think because I will be able to look at it straight away I mean you see people I mean I tend to pick up whatever I am buying and turn it offer and have a good look everting thing if am looking at cooking instruction or where is come from , but if it was in the front quite clear and I could see that it was had a lot of added sugar then that would. "(Participants-WT26) |
|  | coding system needs to be publicized and advertised | "well kind of publicised and advertised otherwise I would not think of colours , if it was kind of on TV and it said this is a new system new colour code too help you choose the right foods for you or something that would kind of put it in your brain."(Participants-WT10) |
|  | Coding system needs to be obvious | " I do like to read labels I do like to understand how this is it’s a red label and it’s say 45 % 45% of what ? and then it’s say it’s daily intake but then it’s in teeny tiny in a font you can’t read so if it was clear I would be happier." (Participants-WT20) |
|  | Picture of tooth decay should be on sugary and fizzy drinks | "of decaying teeth, I think I mean it would have to be like on things like other coca cola or you know really high sugar stuff that you really shouldn’t be having at all, fine" (participant-WT21) |
|  |  |  |
|  | Need for Education about sugar on buses and internet | "yeah I don’t have any educational about sugar [laughing]...ooh yeah yeah definitely, you just need like you know bus stop signs or on the side of the bus or in the internet advertisement just you know information ."(Participants-WT3) |
|  | More campaigns sugar consumption | "because there is no like you talking about government information knowledge you know we have a governments you know campaigns about smoking illness or alcohol abuse or eating too much fat but there is nothing telling me about sugar consumption. "(Participants-WT3) |
|  | GPs should educate the public | " I don’t see much guidance on sugar intake yes off course I say stuff about sugar based health advice it tend to come in the form of diabetes I think worming of about it that kind of thing but I be I need to stop hinge around in different places or pay more attention or state should have or doctors should provide more information so that people are further advised about it. " (Participants-WT18) |
|  | Need for nutritional classes | "if there is kind of nutrition classes offers cause I mean king’s is good with good variety of different training courses, if there is a nutrition one that was offered I would be tempted to do it." (Participants-WT10) |
|  | Need for cheaper prices vegetables | " if fruits and vegetables become cheaper than chocolate crisps and cookies and stuff then I definitely be more attempted to go for the fruits because especially me favourite fruits like cherries and strawberries and they also expensive type so it’s same which easier to just like buy something cheap and not healthy. " (Participants-WT14) |
|  | Need for posters in supermarkets and shops | " yeah within supermarket yeah because you want to know you don’t have to remember how much in all the foods but if there is a post next to the cereal section how sugars in in all the cereals or you know a post in the fruits and vegan section on what fruits have sugar more than others that kind of stuff , that’s easy to recognize you know so you have the information and then you can decide what to buy there and then , I think that would be helpful you don’t have to remember the stuff all the time. "(participant W21) |
|  | Store product layout | “then like right at the front of the store as soon as you go in me store anyway so that influence me to choose that vegetables….the way supermarkets are laid out for example when I going to Morrison’s in north wales where I used to leave, there will be offers for the baked goods so like muffins a pound or buy one and get one free on a pack of flapjacks for example they were very tempting and I did go for it few times and I think offers like that they do influence you.” (participant W7) |
|  | Hunger influence me food intake | "especially if you gone into store and you are hungry already"(participant W7) |
|  | Family Income influences sugar intake | "I think if the food is more expensive than families with low income for example like me or students budget aam wouldn’t buy sugary things even though I don’t buy that much sugar "(Participants-WT7) |
|  | Increase the level of recommended daily sugar intake | " WT21: 5 % sugar is not very much , it depends what , yeah IT: it’s opinion we do respect , so would you give me a figure? WT21: of what I think is high sugar content ? IT: yeah WT21: I don’t know 20 30 % 25 something like that." (Participants-WT21) |
| Social and professional role and identity (Reflective) | Influence Past sugar consumption experience | "aam I did go through phases ages ago trying to cut down sugar and I was amazed how much stuff has so much sugar in it like a bottle of orange juice has like a 50% of sugar intake or something I was just I said it’s impossible. [ laughing] so" (Participant-WT14) |
|  | Influence past experience of healthy cooking | "No almost no because no sweet we buy at all we don’t buy any desserts we don’t buy any cake I mean even if they were in sale I still wouldn’t buy them. I think in past because I like to cook I like to bake so certainly I wouldn’t buy thing like that pre-prepared. ."(Participants-WT19) |
|  | Professional identity influences sugar intake | "aa yeah , as a student you trying to get the cheapest you can yeah. " (Participants-WT16)  "students budget aam wouldn’t buy sugary things even though I don’t buy that much sugar "(Participants-WT7) |
|  | Social identity influences sugar intake | "I am English [ laughing]...we are omnivores we eat anything "(Participants-WT17) |
|  | Individual's personality influences sugar intake. | "yeah , probably if she comes with me she might say let’s try this or try that ...... yeah , I am quite flexible , I think." (Participant-WT16). " I am actually incredibly difficult person to be influenced [laughing] in any ways" (Participant-WT4)   "I am not going to spend me time adding all of those different things up from the labels so I don’t do that I tend to based pure on me what I do know diet and that kind of thing and I suppose it’s Intuition." (Participants-WT18) |
|  | Human nature influence sugar intake | "but you know I am human, now and again if I feeling down I will reach for the chocolate because it’s elevate the mode [laughing]."(Participants-WT4) |
|  | individual social class and political identity influence sugar intake | "I am white middle class liberal I try me food organic food that’s Fairtrade all of the clichés yeah I tried I do that other foods that might be some food I will buy like a budget of brand" (Participants-WT18) |
|  |  |  |
| Beliefs about Capabilities | Belief easy to identify the amount of sugar in food when cooking | "when we make food from the basic ingredients than it’s reasonable easy because if when we make a cake and they say there is 4 pounds of sugar then we know that there is 4 pounds in there." (Participants-WT17) |
|  | Belief to quantify personal sugar intake | "you know I do like me sweety things I try I restricted myself to have pudding rather than snacks whole day and I know a lot of friends who eat a lot of sugar so I guess that how I can quantify it up for myself."(Participants-WT12) |
|  | Belief to find out sugar content in food from the label | "if I look in the labels that I can find some kind of sugar content" (Participant-WT18) |
|  | Belief to understand food labelling in percentage only | "I would prefer percentages .. because they seems easy to understand quickly whereas having to convert you know let’s say you are only allowed 10 grams and you used 3.5 grams in this I don’t know." (Participants-WT19) |
|  |  |  |
|  |  |  |
|  | belief to find the sugar content in food from the packaging | "no it’s I would be you know even think of cooking dinner I have me me three potato and me one pepper and yes I can find the sugar contents of all of these things ." (participant WT25) |
|  | Belief to easily calculate me amount of sugar intake per day | "no it’s I would be you know even think of cooking dinner I have me me three potato and me one pepper and yes I can find the sugar contents of all of these things but I would have to then calculate how much it added up to for example I think for orange juice I have in the morning I can work it out quite easily because what tells me in the pack ." (participant WT25) |
|  | Belief it’s difficult to control sugar intake | " IT: can you control yourself ? WT3: aam not really [ laughing] it’s quite hard it’s really difficult yeah it’s more difficult than you think." (Participants-WT3) |
|  | Belief can't reduce the intake of sweet snacks | " aaam yeah I just I prioritise what I like over the sugar content I think whereas I think you can there are nice meal you can have without sugar. but the snack I really like . IT: you can’t ? WT11: I can’t get ready of them [laughing]"(Participants-WT11). |
|  | Belief it is easy to get sweets | " it’s just so easy to get in and get a bar of chocolate and eat on the tube on the way to home [laughing]"(Participants-WT7) |
|  | Belief being lazy in me food choices | "aamm probably laziness [laughing]. aam I just yeah I just don’t, I think if I care more by myself and what I was eating then I would probably think about it and I iii again I do go through phases where I follow a diet plan or something like that but again it’s rare , day to day I don’t care enough I suppose."(Participants-WT10) |
|  | Belief it is hard to know the sugar content of ready meals | "aaam I mean I rarely I have takeaways but with takeaways I find it impossible to know how much sugar that was"(Participant-WT19) |
|  | Belief it’s difficult to control the influence of advertisements. | "so aam I think I have been exposed to so much television and mainly television but also kind of billboard advertisement for fizzy drinks that it’s almost an subconscious kind of I don’t have control over it any more so or that much control so if hear someone open a can of coco I kind I want a can of coco or if I see someone drink a can of coco I kind want on" (Participants-WT25). |
|  | Belief it is difficult to read food labels | "when I shop if I could see a clear label stating how much sugar it’s actually in the product but yeah it’s not that easy to read at the moment... it’s not easy now because you really have to look for it you kind you have to analyse it so." (Participants-WT20) |
|  | Belief I can't understand food labels | "the truth is even having read about it I don’t’ understand that much about so I would rarely look at the labels" (Participants-WT24) |
| Optimism | Food labelling is the key for reducing sugar intake when buying food for the first time | " so I think labelling would be the key thing aa for me , because I know not everyone does when they go shopping but I do tend to look if something I am buying for the first time , aam so yeah for me personally is the main thing. " (Participants-WT11) |
|  | Colour coding is the key for selecting low sugar food. | " it’s helps definitely I mean not may be not I don’t know about sugar but about pretty much like fats carbohydrates you can see it very clearly and when you pack product and you see all reds you kind of have these like all red you know that a warning sign I should not have this , so definitely it work for a lots of people" (Participants-WT20) |
|  | Colour coding is the key for attracting attention | " I think that’s definitely is the best way so first it draw your attention to it see the red one then if you are interested like I am it actually pick up exactly why it’s actually red that it’s the percentage that how many grams what is in the back of it , oh it’s not just tomato sauce and herbs it’s abet of long list of other things (Participants-WT7) |
|  | Colour coding is the key for food comparison and selection of lower sugar food | "Definitely yeah well I mean you know naturally looking something red it says you know around everywhere it says stop or warning or alert or something so if you see something is green on it I will draw your eyes first to second attention you will look at it and say oh it’s healthy it’s fine and it has some red on it you will know kind of you can compare things better if I am looking at two sets of numbers let say I don’t know chicken breast vs chicken thighs for example or different types of mincemeats if I am sticking at two set of numbers it’s hard to compare them among each other but if say one had all green and one had three green and two yellow then you could quite easily see that the green one is better and in one part so I think it’s very useful." (Participants-WT27) |
|  | Picture of tooth decay on product is the most effective way to reduce sugar. | "I think tooth decay image would be the best because if someone can see it then they you know attract the eyes a bit more as opposed to number or colours I don’t think. in the way that it will scare me in to thinking that you know me teeth will goanna rot way or you know , I want try to avoid as much as possible if I can you know getting replacement teeth for denture when I get older or anything like that".(Participants-WT22) |
|  | Education about sugar is the key for reducing the sugar intake in children. | "IT: education about sugar in food do you think it would help or not ? WT21: yeah massively... in school for kids."(Participants-WT21) |
|  | Education on sugar in food will work for adult | " IT: okay education about sugar in foods or meals do you think if you know or aware sugar in food it will help to select ? WT27: yeah definitely "(Participants-WT27) |
|  | Sugar tax is the key to reduce the sugar intake of the nation | IT: okay government restriction in amount of sugar in food or food tax or sugar tax , do you think it will help you ? WT23: yeap definitly it will help me and it’s certainly will help the nation. (Participants-WT23) |
| Intentions | Intention not to buy sugary foods | "I want to get me physical aa me physic in shape so the food I buy you know I am not going to buy ice cream or crisps " (Participants-WT3) |
|  | Intention to reduce me sugar intake | " I kind of being in me idea of you know cutting down sugar and eating healthy diet I just wanted to try you know this meal" (Participants-WT20) |
|  | Intention to eat healthy food | "I aam like I say I go to the gym so aam I think you are always a bit more conscious felt what you eat after you have been to the gym because it’s just like I have spent so much time and effort and it was so hard I don’t want to thought all away by having something really unhealthy to eat now and also if I know that I can’t go to the gym one day, I probably wouldn’t eat as bad probably make conscious effort to avoid certain foods because I know that I am not exercise them off." (Participants-WT14) |
|  | Intention to buy unhealthy food | " again if you decided to go and buy lunch I have already have decided I already know it’s not be going to a healthy choice I won’t really consider it."(Participants-WT25) |
| Goals | Goal to reduce me weight | "I want to lose weight" (Participants-WT17) |
|  | Goal to get in good physical shape | "I want to get me physical aa me physic in shape so the food I buy you know I am not going to buy ice cream or crisps " (Participants-WT3) |
|  | Goal to gain weight | "aam so because like I play in full as well the aim is to gain weight"(Participants-WT27) |
| Reinforcement | Colour coding reinforces selection of healthy food | "The traffic light it shows you know it’s gives you positive reinforcement if you get something which is all green you got some rice cake or whatever than or anything fill with some harm or chicken or whatever you what" (Participants-WT27) |
|  | Exercise food labelling telling is behaviour rewards | "IT: amount of exercise to burn of energy, so if you have a product and they say if you consume this product you would need to run for half of hour to burn your calories , do you think this will help? WT11: yeah I think that’s quite a good good incentives particularly aam for someone me position who exercises somewhat but not a lot." (Participants-WT11) |
|  | Education about sugar reinforces decisions about reducing sugar intake | "IT: okay , education about sugar in food , do you think education about sugar in food would help you to select lower sugar lower foods, foods with lower sugar? WT25: yeah probably I am not sure how much of influence it will have , it probably reinforce the decision I am making already or might help to make better once yes I think it will have a positive effect." (Participants-WT25) |
| Social and professional role and identity (Automatic) | Sugar intake is habitual response | " Generally speaking I am creature of habit when it come to me shopping I try to buy I again it does depend on the food stuff I try to buy as far as fruits and veges concerns" (Participants-WT18) |
|  | Cooking food every day | "normally every day I make me own lunch and it normally a sandwich." (Participants-WT19) |
|  | Craving of sugar is a habit | "then the chocolate I have I crave it at that time of the day probably because it’s the habit because I pass the same shop and because it taste nice." (Participants-WT7) " I try to get milk, this was always with me since when I was little. it’s more tradition and I love biscuits." (Participants-WT6) |
|  | Daily buy sweets | "then the chocolate I have I crave it at that time of the day probably because it’s the habit because I pass the same shop and because it taste nice." (Participants-WT7) |
|  | Holiday influences sugar intake changes | "I had I started drinking them again when I went on holidays because I thought I am on holiday so it’s fine [Laughing] yeah and got back and I said well it’s still kind of like holiday feelings so I just have may be one and then aam yeah just got back into the habit."(Participants-WT14) |
|  | Habit of sharing foods with others | "I said before that me eating habit tend to be shared so when the biscuits gets opened I am in there too [laughing]"(Participants-WT17) |
|  | Education about sugar in food during childhood develop a good habit for reducing sugar intake. | " aam about habit start in childhood so if you can educate kids about it in younger aaam ."(Participants-WT14) |
| Emotional | feeling of guilty after eating sweets | " I always like I love like kinder Vance all those things though, but after while I feel guilty [laughing]"(Participants-WT6) |
|  | Dislike sugary foods | "aam I don’t ever buy fizzy drink I don’t like them especially aam I don’t like coco the one which is not diet because it leave like often with a lot of fizzy drinks it leaves like a film of roughness on your teeth so don’t drink fizzy drink for that reason" (Participants-WT7) |
|  | Like the taste of hot drinks without sugar. | " I only drink tea (not coffee) and I like the taste without sugar."(Participants-WT11) |
|  | Like the taste of cold drinks without sugar |  |
|  | Like the taste of fruits | " I like the taste of apple." (Participants-WT19) |
|  | Love fruit with a main meal | "yeah I love to have me fruits with me lunch or as a snack I would have it at about in 3 pm."(Participants-WT25) |
|  | Love fruit as snack |  |
|  | Crave fizzy drinks | "so aam I think I have been exposed to so much television and mainly television but also kind of billboard advertisement for fizzy drinks that it’s almost an subconscious kind of I don’t have control over it any more so or that much control so if hear someone open a can of coco I kind I want a can of coco or if I see someone drink a can of coco I kind want on" (Participants-WT25). "fizzy drink I really like fizzy drinks"(Participants-WT12) |
|  | Body craves sweets | " aaam it’s just what I crave aam me body used to crave something sweet and sugary at some point in the day."(Participants-WT14) |
|  | Love drinking Coca Cola | " I love coca cola as it is not coca cola zero because for me it’s different taste than I will not go for coca cola zero, just because for me it’s a different taste."(Participants-WT6) |
|  | Want for tasty foods Occasionally | " if I am going out for a meal I just want something really tasty because it’s a treat you don’t do it very often do you."(Participants-WT10) |
|  | Treats are sweets |  |
|  | Love bargain foods | "I love bargain I love bargain and if something on sale I would be more like to pay attention." (Participants-WT15) |
|  | No interest in counting sugar content of foods | "I have no motivation or interest in calculating sugar content of me food that I prepare myself because I don’t consider to be higher in sugar. "(Participants-WT25) |
|  | A picture of tooth decay on packaging leads to fear of getting tooth decay | "I think tooth decay image would be the best because if someone can see it then they you know attract the eyes a bit more as opposed to number or colours I don’t think. in the way that it will scare me in to thinking that you know me teeth will goanna rot way or you know , I want try to avoid as much as possible if I can you know getting replacement teeth for denture when I get older or anything like that".(Participants-WT22) |
|  | A picture of tooth decay on packaging is annoying | "if me breakfast cereal or I don’t know or if like if me box of orange juice you know that I have one glass every two days has decaying teeth on it’s like a pack of cigarette that would annoying me. because I feel like I am doing something wrong you know like just just I don’t need someone to tell whether or rather I should be having a glass of orange juice." (Participsnt-WT21) |
|  | Picture of tooth decay on packaging lead to guilty feeling. | "..but if you had on you know a single biscuit it might be a bit too much or make me feel really guilty if I have any sugar at all it’s fine moderation ."(Participants-WT27) |
|  | Tooth decay image on packaging leads to have no autonomy in a decision. | "if me breakfast cereal or I don’t know or if like if me box of orange juice you know that I have one glass every two days has decaying teeth on it’s like a pack of cigarette that would annoying me. because I feel like I am doing something wrong you know like just just I don’t need someone to tell whether or rather I should be having a glass of orange juice." (Participsnt-WT21) |
|  | Tooth decay image on packaging is horrible. | "will be image of the tooth decay because that. it would be most effective but it will be horrible to be in the supermarket with images of physical native things." (Participants-WT3) |
|  | Enjoying eating sugary snacks | "I snack a lot of sugars and biscuits it was fun I had a fun when I think of biscuits" (Participants-WT7) |
|  | Negative experience with fitness apps | "When you do me fitness pal you are trying to be healthy aam I got to the point where I was like I am actually going to ignore it what it tells me about sugar because it just went it was away over for me you know I cancel the fruits and things that counted towards I would know aam I could have as many bananas [laughing] and apples that I could and it didn’t matter and may be only keep cut of the sugar and stay something like me muesli bar or me soya milk and things like that with added" (Participants-WT15) |
| Beliefs about Consequence | Belief that low calorie food are healthy | "aam amm I would say I usually go for , it’s healthy, there is usually one under 500 calories and one is over, so on the days that they have one under 500 calories I really like the look of it I will have that because it’s the healthy option again if there is something I don’t like either of the options I will go for jack of potato, just because again I don’t really like it, but it’s healthy and I don’t like the other two things okay they have." (Participant-WT14) |
|  | Belief that sweets are not very healthy | "chocolate and chocolate bars and things like that normally is artificial sugar that’s’ not very healthy for you so accept. and the good sugar fructose and all the you know natural occurring sugar is in fruits and vegetable then to be perceived as more healthy so yeah me understanding of sugar is definitely."(Participants-WT20) |
|  | Belief that natural occurring sugar is healthy. |  |
|  | Belief that cooking food has healthy consequences | " I cook quite a lot try to be healthy" (Participants-WT11) |
|  | Belief that fruits with high sugar is bad for teeth | so I think, fruits with high sugar content aam sweets with high in sugar content also I think carbonate drinks are bad for your teeth aam. "(Participant-WT25) |
|  | Belief that exercise helps to consume less sweet food | " yeah I think if aam on a day when I done exercise aam it can go either way, either if you feel good about having exercise and so more healthy and avoid snack ....." (Participants-WT11) |
|  | Belief that performing exercise without reducing sugar intake is healthy | "I am trying to lose weight by doing more running and do more fitness exercise so I still eat the same amount of food but I just increase amount of exercise." (Participants-WT3). |
|  | Belief that consuming sugar is fine as long as doing exercise | " IT: you told me that you parents also told you that is fine if you are young to have sweets. WT16: yeah yeah well in a way I mean if you going to burn it off then it’s okay."(Participants-WT16). |
|  | Belief that drinking a lot of fruit juice reduces illness. | " aam so if I ever get if start get cold or feel a bit ill I will always drink a lot of apple juice or eat some oranges juice or take some vitamins or something like that, because I think it might help but I might be too late and then I have to get" (Participants-WT13). |
|  | Belief that drinking fizzy drink has positive consequences | "I will have fizzy drinks if I am sleepy may be and just want a little boost" "I think but I know that in moderation these products that high sugar is fine already I belief it’s fine and I belief that I am using in moderation" (Participants-WT25) |
|  | Belief having sugar in moderation is okay | "I think but I know that in moderation these products that high sugar is fine already I belief it’s fine and I belief that I am using in moderation" (Participants-WT25) |
|  | Belief exercise increases snacking | " ....... or else you feel like you done exercise therefore you can have more like cake and biscuits and things, so definitely it does make difference and I am conscious of falling between sport and what I eat but it’s quite unpredictable so." (Participants-WT11) |
|  | Belief low sugar foods taste bad | "I think if it’s taste , will put it this way I don’t see any point in having aam say low fat low sugar yogurt if it doesn’t taste anything" (Participant-WT26) |
|  | Belief only high fat and high calorie foods are bad for me | " I tend to focus on like the calories and fat saturated fats because I know that it’s bad for you as well. " (Participant-WT14) |
|  | Belief food labels in percentage of sugar is helpful in reducing sugar intake | "yeah I think so I think in percentage it would be a lot better. .. aam so if I pick up a chocolate bar and said that it has got a 30% of your daily sugar probably I will put it start back down and thinking that’s really bad , I mean they might do it now but I don’t know (Participants-WT13) |
|  | Belief food labels in grams of sugar is helpful in reducing sugar intake |  |
|  | Belief food labels in grams and percentage of sugar is helpful in reducing sugar intake |  |
|  | Belief food labelling in grams and percentage helpful with ready meals | "IT: do you think amount of sugar in food amount of sugar in percentage and grams would help you.? WT16: aam yeah probably for the sweeter things. but It wouldn’t really help if you are dinner having like aam a beef curry or something it wouldn’t be very helpful but if it you are having a doughnut it might be , you might think more about. " (Participants-WT16) |
|  | Belief food labelling in grams and percentage helpful with sugary foods e.g. sweets. |  |
|  | Belief presenting sugar content in percentage or grams are helpful in reducing sugar intake when comparing two products together | "IT: labels amount of sugar in food do you thing the amount as percentage or grams do you think it will help you I selecting foods with lower sugar? WT11: aaam not unless if I was comparing it with something else just a number by itself it wouldn’t." (Participants-WT11) |
|  | Belief colour coding system leads to selection of low sugar food by attracting your attention. | "IT: yes do you think this would help you to select low sugar? WT27: definitely yeah, well I mean you know naturally looking something red it says you know around everywhere it says stop or warning or alert or something so if you see something is green on it I will draw your eyes first to second attention you will look at it and say oh it’s healthy it’s fine and it has some red on it you will know kind of you can compare things better if I am looking at two sets of numbers let say I don’t know chicken breast vs chicken thighs for example or different types of mincemeats if I am sticking at two set of numbers it’s hard to compare them among each other but if say one had all green and one had three green and two yellow then you could quite easily see that the green one is better and in one part so I think it’s very useful." (Participants-WT27)   "Definitely for example with the jars what I have mentioned quite a lot jars with sauces I stopped choosing that because there was the traffic light labelling system on it which told me exactly how much was in there, so the labels very helpful. " " I think that’s definitely is the best way so first it draw your attention to it see the red one then if you are interested like I am it actually pick up exactly why it’s actually red that it’s the percentage that how many grams what is in the back of it , oh it’s not just tomato sauce and herbs it’s abet of long list of other things"(Participants-WT7) |
|  | Belief colour coding system leads to selection of low sugar food by allowing comparison between food products |  |
|  | Belief that colour coding leads to selection of low sugar food assessing the content of the products |  |
|  | Belief colour coding leads to more awareness of food choice. | " I find the colour coding is the most informative .. on which highlight the facts of how bad it is "(Participants-WT23) |
|  | Belief colour coding won't reduce sugar intake for a normal person. | "I guess it’s abet analogous like the whole smoking situation where they put how bad it’s for your lungs in the package and smokers still buy the cigarettes and I think it’s the same situation I think like until really you have something wrong with you know until you get diabetes actually you don’t take it in consideration aam and I think that the problem with sugar a lots of people including myself we see that in there front they have you know red sticker for like sugar and fat but you still end up buying it so it doesn’t really put me off at all. " (Participants-WT22) |
|  | Belief colour coding won't reduce sugar intake for a normal person. |  |
|  | **Belief colour coding is not useful when buying or comparing products that have the same sugar content.** | "I don’t know I think it will be less useful when you buying if you say I went out to get a chocolate bar if they all red there is not much comparison you can’t tell compared with within the chocolate bar which one is the worse ." "it might do aam I guess if there if you have I mean if I was going out to buy a sweet bar a bar of chocolate , there all going to be red so there won’t be much sort of different between the bars, you get what I mean. (Participants-WT16) |
|  | Belief that colour coding is a very poor way of presenting information | " no I don’t think so because I, you could end up with an awful lot of information on a page on the front in the base so and also if it’s says the red and green that doesn’t always help, but for me I think I just want the information whether it says green or red that’s me I think it’s for me to decide whether I think it’s." (Participants-WT26) |
|  | Belief an amount of exercise to burn off energy label leads to thinking about sugar consumption before eating. | " yeah it puts in perspective like I know aam because I do that for myself I know that for example a package of Maltsters is half an hour on exercise bike which is actually quite long time. so if you are more aware of it more people aware it then I think it wll make people to reconsider what they eat because you I think you realise how much hard work it takes to burn something like that off. " (Participants-WT14) |
|  | Belief an amount of exercise to burn off energy label increases awareness of how much exercise you need to do to burn off energy. | " I think it will be an interesting one aam because I have only very recently myself looking at very roughly that sort of thing I know when I am at the gym it’s a great because I am using a machine and it will tell me okay I you have just burn a 100 calories ten minutes on the treadmill whatever it is and to be able associated with it yes that could be help may be." (Participants-WT15) |
|  | Belief that an amount of exercise to burn off energy label helps to control sweet snacks | " is actually because now I am going to the gym it’s actually make me think about the way about what I am eating in different way but I don’t know whether that would necessary stop me from eating I don’t I think it would help to me may be control some of other things I eat may be like bar of chocolate for example after the gym"(Participants-WT7) "yeah I would love that. if I have a chocolate bar and they say you have to walk like an hour to burn this off , yeah...because it would make me awake , because I think a lot of people and I do too you just kind you eat and you think ooh that not that’s bad ooh that not that’s bad and then things kind of stuck up whereas if you want ooh this chocolate bar I have to do that much exercise to burn off you then do that much of exercise you like okay so now I am back to a level I have eaten chocolate bar but I hasn’t kind of negatively affected me. "(Participants-WT13)  "because you might just say oh who cares I will eat it anyone and I will run for 20 minuets do you see what I mean. it might not be the like but because I go to the gym quite regularly so I know that if I want to eat something in fact I do that now I don’t really control what I eat I just exercise more if I am going to have like bad weekend or something you know if I am have a massive meal one night the next day I probably will go to the gym for longer or something like that you know."(Participants-WT21) |
|  | Belief that an amount of exercise to burn off energy label encourages healthy meals. | "possibly the exercise one well I am in two minds about it, on one hand it will be quite interesting to see you can either eat this per or you can’t eat whatever this is and exercise 60 minutes to burn it off or you could choice something healthier aam" (Participants-WT10) |
|  | Belief that an amount of exercise to burn off energy label will not influence the choices of a healthy person | "yeah I don’t think, I think the problem with me I have always have quite good metabolism I am not sure how damage it will be doing, but internally to me because I don’t see it in the outside but I am sure for other people would work you know if you telling them you know you need to do this amount of exercise to burn this amount of calories from what you have consumed but I think for me personally because I have not had any issue of aam it pro won’t work for me."(Participants-WT22) |
|  | Belief amount of exercise to burn off energy label is not useful for people who are not performing exercise. | "if says you need to run 100 meters I wouldn’t know how to calibrate that with what I do , so it might be of some people but I don’t do any running or any formal exercise I just make sure I do a lot of walking , aam it will be less relevant to me. "(Participants-WT18) |
|  | Belief placing a picture of tooth decay on food packaging is acceptable on a sugary drink. | "....you won’t be desensitized to it if it’s really bad I don’t know like like ginger beer or something or coca cola may be it could be good but you have to start some threshold I think ."(Participants-WT27) |
|  | Belief placing a picture of tooth decay on food packaging will help to reduce the need for dental treatment | "I think tooth decay image would be the best because if someone can see it then they you know attract the eyes a bit more as opposed to number or colours I don’t think. in the way that it will scare me in to thinking that you know me teeth will goanna rot way or you know , I want try to avoid as much as possible if I can you know getting replacement teeth for denture when I get older or anything like that".(Participants-WT22) |
|  | Belief placing a picture of tooth decay on food packaging will reduce sugar intake | "it will put me off. "(Participant-WT14) |
|  | Belief placing a tooth decay image on food packaging is acceptable in sugary food | "of decaying teeth, I think I mean it would have to be like on things like other coca cola or you know really high sugar stuff that you really shouldn’t be having at all, fine " (Participsnt-WT21) |
|  | Believe placing a picture of tooth decay on food packaging has no influence on an individual with no dental decay | "IT: okay alright what about image of dental caries , a tooth with caries, in product , do you think it will help ? WT11: aamm I think it will be kind of piercing but personally it wouldn’t because they haven’t had any caries it wouldn’t resonate me personally it wouldn’t make me think if I eat that I am going to be that so it won’t be nice thing to look at aam but I don’t think it will make any difference if I consume that product or not for me personally." (Participants-WT11) |
|  | Belief placing a picture of tooth decay on food packaging will be rejected by the public | " but I am not sure if it will be you know welcomed I"(Participants-WT27) |
|  | Belief placing a picture of tooth decay on food packaging will leads to feeling guilty. | "maybe I mean I don’t know I don’t know may be I am not sure what kind of food that cause obviously things like smoking are really bad for you you know even if in small amount if you have one cigarette a day still bad for you and there is image are helpful to stay away , but if you had on you know a single biscuit it might be a bit too much or make me feel really guilty if I have any sugar at all it’s fine moderation."(Participants-WT27) |
|  | Belief that education about sugar in food will lead to good results for body and health | " but if have the knowledge about 50% of sugar is bad or 20% you know if have the understanding of sugar and the impact on you know physic, weight , health, fitness than I would definitely take note of you know labels and for me personally percentage will be more effective than colour code."(Participants-WT3) |
|  | Belief that education about sugar in food enables to make judgement on sugar intake. | " yes I think would aam I think especially for me realise the different between or perhaps need information about the difference between the different types of sugars amm but then you get all education knowledge about what you are eating and how it all what’s there I think is all in the sugar it is useful .I think enable to make judgement on again test whether the sugar is is part of the item that I am buying or has been added by the manufactures I and I do like to know what’s has sort of part in to it afterwards yeah, so." (Participants-WT26) |
|  | Belief that education about sugar in food helps to decide on healthy food | " yeah probably I am not sure how much of influence it will have , it probably reinforce the decision I am making already or might help to make better once yes I think it will have a positive effect.". (Participants-WT25) |
|  | Belief that education about sugar in food has no effect | " aaam I think I don’t think I am I don’t think I know everything about it by any means, but I think relative to the average I am fairly well informed am so I am not sure that that education particularly help me because although I know the risks of consuming a lot sugar still I do , despite that because I prioritizing taste as suppose over the health health risk, I think education is really important over all but for me I don’t think it will make a big difference."(Participants-WT11) |
|  | Belief that a sugar or food tax helps the nation to reduce sugar intake. | IT: okay government restriction in amount of sugar in food or from food tax or sugar tax , do you think it will help you ? WT23: yeap definitly it will help me and it’s certainly will help the nation. (Participants-WT23) |
|  | Belief that sugar or food tax reduces the frequency of sugar intake. | " suppose me chocolate bar was a pound then I probably think about less a week for example may be to two times a week as opposed to three times a week yeah I think that a very good point that if it’s more expensive if it was taxed so that would increase the price, wouldn’t it ". (Participants-WT7) |
|  | Belief that high sugar tax reduces sugar intake only | " if it was 20% from 50p to I suppose that still 60 p then you know it would start to notice, obviously the problem that keep people saying is that that target that poor people attacks on poor people and probably will still buy it but in less so I mean because I already know I am not sure how it will work but it’s a good idea "(Participants-WT27) |
|  | Belief that high sugar tax has no effect for people with low sugar consumption | " I think that it, personally I don’t think it will affect me too much I don’t feel I have a high sugar diet "(Participants-WT17) |
|  | Belief that high sugar tax has no effect on reducing sugar intake. | " it’s hard to say how much tax it would like how much tax it would be there is 5% tax if you have a chocolate bar that went from 50 p to 55p I don’t think people would care that much , I am not against I would say I am for sugar taxing but you know because I am not eating a lots of sugar it won’t make difference I suppose because you know if you count coke or whatever it was 60p and then it was a pound suddenly , it doesn’t seems important , whereas like good to buy." "(Participants-WT27) |
|  | Belief that high sugar tax leads to selecting the cheapest sugary food | " aam I think so well , I don’t think , so it never gone be sort of a huge amount they put one , so I think if I really want a chocolate bar or fizzy drink I just pay the extra , I don’t think it will ever really determine me , but it will make me may be think about it a bit more if they were two drinks I like and one has an extra 20 pens and the other one a bit cheaper okay this may be better for me I will take that one instead. "(Participants-WT13) |
|  | Belief that labelling packaging with sugar amounts against the recommended level of intake is helpful in selecting food with low sugar. | " IT: so because currently in every product there is only amount of sugar with it only do you think this would help ? WT19: I think to have it against the recommended limits definitely help. " (Participants-WT19) |
|  | Belief TV advertisement on low sugar intake will help in reducing sugar intake | "me and me boyfriend were talking on this the other day actually, that do you know the adverts the government put on the move more exercise move more adverts like cartoon character I think it’s something life or something. there was   more adverts on the TV aimed at people not sell them stuff ,  to tell them this is how much sugar  is in this just education , so people everyone watches TV pretty much everyone watches TV so if you put on an adverts prime time where everyone going to  see it and it’s going to be shock facts like what you said about  tooth decay , having a picture of tooth decay associated  with a chocolate bar that kind of thing , this is how many is this will that you have to do to burn off this  chocolate bar, or this ready meal Bolognese’s ready meal which doesn’t look like it has sugar on it actually contains this many cup of sugar do you that kind of things,   more kind of these  adverts  are  very  helpful and would be a good way to educate the public especially something called Talent show ,something everyone going to watch a whole diverse range of people kids to old people  as going to watch that show  if  it’s on   the adverts on there  then that’s going to capture a wide range of people attention, that’s." (Participants-WT7) |
|  | Belief that the percentage of individual 's daily intake is useful in helping people to think about the food before buying it | "so I think the percentage of the content would be more like a point of interest GP like ooh okay whereas the percentage of your daily is more likely to inform you the choices so if you knew that by eating this type well this 50% of something for day then you might think twice about it. this what I want me 50% to be or something else that I want more , aaam so I think the percentage of your dial would be the most useful if you trying to reduce your intake" (Participants-WT11) |
|  | Belief obvious labelling system helps careful thinking of foods with high sugar and daily food plan | " aam so if I pick up a chocolate bar and said that it has got a 30% of your daily sugar probably I will put it start back down and thinking that’s really bad , I mean they might do it now but I don’t know , but I think sort of to make it clearer and more obvious I think I would think more careful about having certain things that have high sugar in and think how would else for me eating for the rest of the day."(Participants-WT11) |
|  |  |  |
